# Determinants of Continuous Smartwatch Use and Data-Sharing Preferences With Physicians, Public Health Authorities, and Private Companies: Cross-Sectional Survey of Smartwatch Users

**DOI:** 10.2196/67414

**Published:** 2025-08-18

**Authors:** Anthony James Goodings, Kayode Philip Fadahunsi, Derjung M Tarn, Jennifer Lutomski, Allison Chhor, Frances Shiely, Patrick Henn, John O'Donoghue

**Affiliations:** 1 School of Medicine, University College Cork, Cork, Ireland; 2 Department of Family Medicine, Memorial University of Newfoundland, Fogo Island, NL, Canada; 3 Department of Primary Care and Public Health, Imperial College London, London, United Kingdom; 4 David Geffen School of Medicine at UCLA, University of California, Los Angeles, 10880 Wilshire Blvd, Suite 1800, Los Angeles, CA, 90024, United States, 1 (310) 794-8242; 5 Department of Emergency and Critical Health Care, HAN University of Applied Sciences, Nijmegen, The Netherlands; 6 Faculty of Medicine, University of Ottawa, Ottawa, ON, Canada; 7 HRB Clinical Research Facility and School of Public Health, University College Cork, Cork, Ireland; 8 Malawi eHealth Research Centre, Mzuzu University, Mzuzu, Malawi; 9 Business Information Systems, University College Cork, Cork, Ireland

**Keywords:** smartwatches, wearable electronic devices, health behavior, privacy, confidentiality, user engagement, digital health, perceived enjoyment, user satisfaction, data anonymization, continuous use, telemedicine, smartwatch, smartwatch use, preferences, physicians, public health authorities, private company, surveys, users, cross-sectional, online survey, expectation-confirmation model, structural equation modeling, wearable technology, wearables, data sharing

## Abstract

**Background:**

Smartwatches are widely adopted globally for tracking health metrics, offering potential for enhancing individual health care and public health efforts. Continuous use of the devices and users’ willingness to share the data collected are critical to realizing their full benefits.

**Objective:**

This study aimed to identify key factors that determine continuous smartwatch use and users’ comfort levels in sharing health data with health care providers and public health authorities.

**Methods:**

A cross-sectional online survey of current and past smartwatch users (aged >18 years) was conducted to assess determinants of continuous use based on the Expectation-Confirmation Model (ECM) and user comfort levels with different data-sharing methods. Structural equation modeling was used to evaluate relationships between habit formation, satisfaction, perceived enjoyment, and perceived usefulness with continuous use. Wilcoxon signed-rank tests were used to analyze user comfort in sharing data, comparing noninternet- versus internet-based sharing methods and fully versus partially anonymized data.

**Results:**

A total of 273 responses were analyzed, with participants aged 18‐65 (mean 35.6, SD 11.7) years. The results indicate that continuous use of smartwatches is explained by habit (*β*=.35; *P*<.001) and satisfaction (*β*=.38; *P*<.001), which is in turn explained by perceived usefulness (*β*=.38; *P*<.001), perceived enjoyment (*β*=.32; *P*<.001), confirmation (*β*=.24; *P*<.001), and perceived usability (*β*=.10; *P*=.03). Smartwatch users preferred noninternet-based sharing options (z=−5.793; *P*<.001) when sharing data with their physician. Similarly, users were more comfortable sharing fully anonymized data with public health authorities than partially anonymized data (z=−3.592; *P*<.001).

**Conclusions:**

Habit formation and satisfaction emerged as pivotal drivers of continuous intention to use smartwatches, emphasizing the need for features that foster integration into daily routine and a rewarding user experience. Preferences for noninternet-based data sharing with physicians highlight privacy concerns that must be addressed to build users’ trust. By aligning device features and data-sharing protocols with user preferences, manufacturers, health care providers, and policy makers can enhance user engagement and maximize the potential of smartwatches to support individual health management and public health initiatives.

## Introduction

### Background

Based on recent estimates from 2023, there are 5.3 billion internet users and nearly 5 billion social media users worldwide [1], which equates to nearly two-thirds of the global population. This technology explosion is driving a paradigm shift in health care, facilitating patients’ ability to monitor their health from home, engage with fellow patients via social media, and have video consultations with care providers [2]. Such transitions have only been accelerated in the wake of the SARS-CoV-2 pandemic as health care providers seek novel solutions to enhance clinical care. Data collection via smartwatches and other personal devices offers a timely and relevant option.

Smartwatches are a widely used tool for tracking health metrics, enabling users to monitor health metrics, including heart rate, blood oxygen saturation, and conduct single-lead electrocardiograms [3]. Smartwatches contain a selection of sensors that hold potential for enhancing individual health care [4], collecting clinical trial data, and contributing to broader public health efforts. By 2020, one in 5 Americans had used a smartwatch [5], highlighting their growing acceptance and integration into daily routines.

The continuous use of smartwatches and users’ willingness to share the health data collected are essential to maximizing their benefits. Ongoing use ensures a comprehensive and continuous dataset, crucial for monitoring chronic conditions, detecting acute health changes, and supporting long-term health management, such as the development of large language models, machine learning, and artificial intelligence for both public and individual care services. Various factors influence whether users maintain regular engagement with their smartwatches, with enjoyment and habit formation being particularly impactful [6,7]. Further work has established that several factors, including utilitarian, emotional, and health-related reasons, play significant roles in the continued use of smartwatches [8]. Users tend to view their smartwatches as personal and enjoyable devices rather than strictly medical tools [9].

Digital health interventions have continued to show promise as effective and economical interventions [10,11]. Yet passively collected data from smartwatches remains largely unused and may offer rich data to clinicians, public health authorities, and private industry.

An equally critical aspect is a user’s level of comfort with sharing their health data with health care providers. The sensors embedded in these devices generate valuable and sensitive data [3,12,13] that have the potential to improve health care outcomes by providing practitioners with detailed, longitudinal, and up-to-date health information. The serial data contained within these datasets may hold significant value in terms of medical treatment and understanding population characteristics in the context of public health. To establish such datasets, the user must continuously use their device over time. Comfort levels in sharing this data may vary based on the sharing method, but this has remained largely unexplored. Concerns regarding privacy, data breaches, and trust in data-handling entities play crucial roles for end users in shaping their comfort levels [14,15].

Data sharing in health care has been investigated through several studies [16-19]. However, as technology continues to evolve and play a more dominant role in society, it is reasonable to expect attitudes to change. This highlights the importance of continued work in the field; not only have people become more accustomed to technology, but more data are being collected from personal devices than ever before [16,17]. A study from the mid-2000s examining the attitudes toward sharing of health data indicates that the recipient of the data, the level of anonymity, and the type of information are foundational factors [18].

Patients now hold vast amounts of their own health data stored on personal devices and online services. Historically, before the mainstream adoption of smart devices, most health data were kept by health services as medical records. Smart devices now hold an additional set of longitudinal health data outside of medical records. The impact of this on data sharing preferences is largely unknown.

With the widespread success of smartphones and mobile health apps in the 2010s, the general public began to appreciate the large amount of data they hold, and there has been heightened awareness surrounding data privacy [19]. A 2016 qualitative study on the topic highlighted that users saw the data on calories and exercise collected in apps to be private matters [20]. Among concerns was the potential exploitation of data by third parties (eg, insurance companies) [20].

The concern of data misuse is well-founded. Internet users face a near-continuous bombardment of tailored advertisements [21] and even automobiles that collect driving data, which can be sold to insurance companies [22]. To appreciate the potential benefit of the health data collected from smartwatches in health care, it is crucial to understand both the users’ concerns and the reasons behind them.

### Aims and Objectives

This study seeks to examine the key factors that determine continuous smartwatch use and users’ comfort levels in sharing their health data collected from a smartwatch with health care practitioners and public health authorities. By identifying these factors, strategies may be developed to enhance user engagement and data sharing, ultimately improving the integration of smartwatch sensor data into health care practices and public health authorities. As smartwatches are a rapidly evolving technology, it is crucial to re-establish the factors influencing continuous smartwatch use. This paper presents findings from an online survey of smartwatch users, offering insights into the key factors influencing their behaviors and preferences.

By identifying these factors, strategies may be developed to enhance user engagement and data sharing, ultimately improving the integration of smartwatch sensor data into health care practices and public health authorities. As smartwatches are a rapidly evolving technology, it is crucial to re-establish the factors influencing continuous smartwatch use. This paper presents findings from an online survey of smartwatch users, offering insights into the key factors influencing their behaviors and preferences.

## Methods

### Study Design and Setting

This study is a cross-sectional online survey of smartwatch users. The survey was designed to assess the determinants influencing continuous use of smartwatch sensors and users’ comfort levels in sharing health data with health care practitioners and public health authorities. The paper was prepared in line with the STROBE (Strengthening the Reporting of Observational Studies in Epidemiology) guidelines [23] (the STROBE checklist is provided in Checklist 1).

The survey was conducted via the internet using the Qualtrics (Qualtrics LLC) survey platform. Recruitment and data collection were conducted between July and November 2023.

### Participants

The inclusion criteria required participants to be aged 18 years or older and current or past users of smartwatches. Participation was voluntary, and no incentives were offered. Participants were recruited via convenience sampling through various online platforms, including social media (eg, LinkedIn [LinkedIn Corp]), university networks (University College Cork), and community forums (eg, Reddit [Reddit Inc]).

### Data Sources

**Figure 1.**
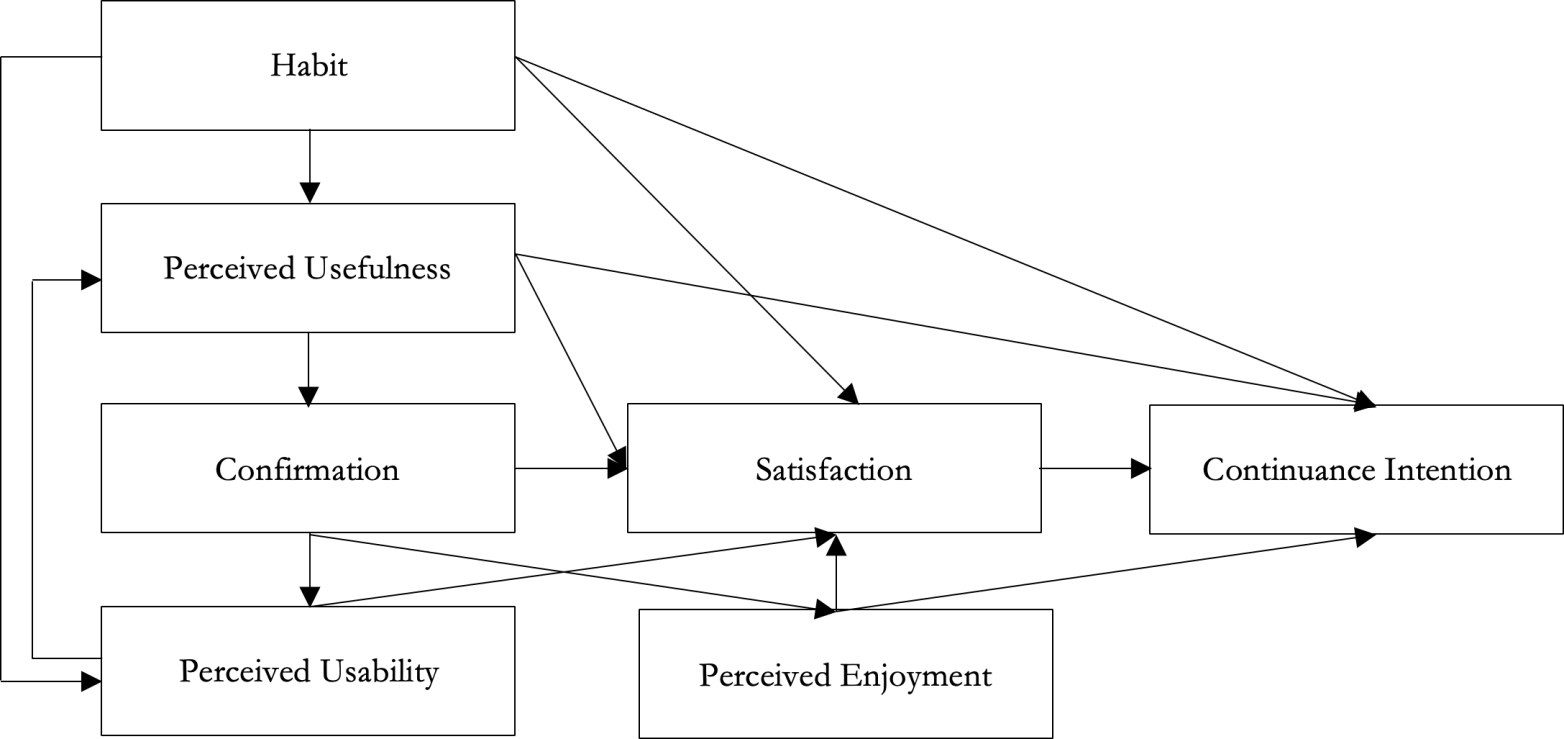
Expectation-Confirmation Model. Adapted from Nascimento et al [6].

The survey consisted of 2 main sections. The first section focused on factors influencing continuous use of smartwatches, based on the Expectation-Confirmation Model (ECM), as shown in Figure 1 [6,24]. The ECM is a widely accepted and validated model that has been used in similar studies, focused on continuous intention to use consumer electronics [6,25]. The first section included 2 items measuring confirmation; 3 items for enjoyment and habit formation; 4 items for perceived usefulness, satisfaction, and continuous intention; and 8 items measuring perceived usability. These were measured on a 7-point Likert scale. For example, perceived enjoyment was assessed with 3 questions: (1) “I have fun interacting with the smartwatch,” (2) “Using the smartwatch provides me with a lot of enjoyment,” and (3) “I enjoy using the smartwatch.” For each question, respondents were asked to choose from a 7-point Likert scale ranging from “entirely disagree” to “entirely agree.” All the questions and response options are included in Multimedia Appendix 1. Each question is coded to the construct it is evaluating, corresponding to Tables1  2. Thus, the first section of the survey is a validated questionnaire that has been used in previous studies [6,25]. Continuous intention is defined as the intention of the user to engage in regular, habitual use of the device for the foreseeable future. Perceived usefulness is defined as the user’s perception of the utility and value derived from the possession and use of the device. Generally, constructs refer to user-endorsed feelings, experience, and perceptions regarding habit formation, satisfaction with the device, perceived enjoyment of using the device, perceived usability of the device and its features, and confirmation of these perceptions.

The second section addressed comfort levels in sharing health data, concentrating on different data-sharing methods (eg, internet vs noninternet) and included items assessing preferences for various methods and concerns about privacy and trust. This section measured responses on a 5-point Likert scale ranging from “extremely comfortable or confident” to “extremely uncomfortable or unconfident.” We use the term “comfort level” to refer to the respondent’s self-reported comfort in sharing smartwatch health data as outlined in each particular scenario presented in the survey. Refer to Multimedia Appendix 1 to view the survey in its entirety.

**Table 1.** Composite reliability (CR), Cronbach α (CA), average variance extracted (AVE), square root of AVE, and correlations.

Construct	Mean (SD)	CR^a^	CA^b^	AVE^c^	CONF^d^	ENJ^e^	HAB^f^	INT^g^	PU^h^	SAT^i^	USAB^j^
CONF	5.37 (1.09)	0.79	0.47	0.65	0.81	—	—	—	—	—	—
ENJ	5.28 (1.08)	0.94	0.90	0.83	0.39	0.91	—	—	—	—	—
HAB	5.75 (1.22)	0.91	0.85	0.77	0.46	0.48	0.88	—	—	—	—
INT	6.41 (1.00)	0.98	0.97	0.93	0.38	0.46	0.63	0.96	—	—	—
PU	5.42 (1.19)	0.91	0.87	0.72	0.50	0.53	0.67	0.46	0.85	—	—
SAT	5.89 (0.93)	0.94	0.92	0.80	0.60	0.67	0.69	0.64	0.72	0.90	—
USAB	5.50 (1.09)	0.92	0.91	0.61	0.46	0.54	0.53	0.35	0.52	0.58	0.78

a CR: composite reliability.

b CA: Cronbach α.

c AVE: average variance extracted.

d CONF: confirmation.

e ENJ: perceived enjoyment.

f HAB: habit.

g INT: continuance intention.

h PU: perceived usability.

i SAT: satisfaction.

j USAB: usability.

**Table 2.** Factor loadings and cross-loadings of the measurement model.

Construct	CONF^a^	ENJ^b^	HAB^c^	INT^d^	PU^e^	SAT^f^	USAB^g^
CONF1	0.867	0.357	0.433	0.378	0.474	0.557	0.392
CONF2	0.741	0.260	0.288	0.218	0.314	0.397	0.357
ENJ1	0.315	0.907	0.379	0.382	0.473	0.580	0.494
ENJ2	0.313	0.929	0.433	0.394	0.480	0.596	0.432
ENJ3	0.422	0.902	0.497	0.465	0.502	0.639	0.543
HAB1	0.393	0.419	0.921	0.655	0.579	0.646	0.436
HAB2	0.441	0.413	0.921	0.604	0.557	0.638	0.453
HAB3	0.368	0.441	0.786	0.371	0.634	0.539	0.487
INT1	0.394	0.434	0.627	0.964	0.488	0.625	0.350
INT2	0.362	0.456	0.594	0.974	0.455	0.641	0.329
INT3	0.320	0.483	0.579	0.974	0.441	0.625	0.339
INT4	0.392	0.388	0.607	0.943	0.387	0.587	0.315
PU1	0.494	0.373	0.631	0.554	0.781	0.658	0.411
PU2	0.368	0.445	0.512	0.259	0.869	0.535	0.416
PU3	0.401	0.488	0.540	0.347	0.882	0.569	0.450
PU4	0.406	0.510	0.565	0.350	0.866	0.647	0.465
SAT1	0.512	0.589	0.650	0.618	0.662	0.911	0.478
SAT2	0.560	0.610	0.618	0.585	0.676	0.922	0.514
SAT3	0.479	0.581	0.560	0.480	0.529	0.818	0.486
SAT4	0.595	0.605	0.652	0.608	0.689	0.923	0.585
USAB 1	0.324	0.387	0.320	0.153	0.320	0.334	0.650
USAB 2	0.280	0.377	0.267	0.141	0.280	0.328	0.622
USAB 3	0.252	0.438	0.322	0.213	0.330	0.360	0.756
USAB 4	0.371	0.410	0.389	0.313	0.440	0.479	0.799
USAB 5	0.322	0.408	0.406	0.237	0.398	0.431	0.837
USAB 6	0.421	0.463	0.445	0.307	0.432	0.513	0.882
USAB 7	0.385	0.437	0.428	0.212	0.403	0.465	0.870
USAB 8	0.458	0.436	0.560	0.455	0.514	0.579	0.763

a CONF: confirmation.

b ENJ: perceived enjoyment.

c HAB: habit.

d INT: continuance intention.

e PU: perceived usability.

f SAT: satisfaction.

g USAB: usability.

### Data Collection

Data were collected using the Qualtrics online survey platform, ensuring anonymity and confidentiality of responses. The survey was open for responses for a period of 3 months, aiming to capture a diverse sample of smartwatch users. An open link was used to distribute the survey. To prevent false responses, tools offered by the survey platform, such as “Bot-Detection” and “reCAPTCHA,” were enabled.

### Study Size

The survey aimed to recruit approximately 200 participants, as recommended in the literature, to provide sufficient power for the model to yield a meaningful analysis to reduce the impact of random error in structural equation modeling (SEM) [26].

### Data Analysis

Data were analyzed using SmartPLS (version 4.1.1.1; SmartPLS GmbH) and Python (version 3.10; Python Software Foundation). Descriptive statistics, including means, SDs, and frequencies, were used to summarize participant demographics and overall response patterns. Inferential statistical tests were used to examine relationships between variables relevant to continuous smartwatch use.

For the analysis of factors influencing continuous smartwatch use, SEM was conducted using SmartPLS version 4.1.1.1. The SEM analysis was used to assess the reliability and validity of the ECM and test the relationships among the constructs, including habit formation, perceived enjoyment, satisfaction, perceived usefulness, confirmation, perceived usability, and continuance intention. A bootstrapping procedure was applied to assess the significance of path coefficients.

For the analysis of the sharing health data section, Python was used with key packages including “pandas” for data manipulation, “numpy” for numerical operations, “scipy” for statistical testing, and “matplotlib” for data visualization. Wilcoxon signed-rank tests were used to compare paired data, reflecting respondents’ comfort under different sharing scenarios. The first comparison assessed comfort with sharing data with a physician via noninternet methods (eg, wired connection, Bluetooth, and near-field communication [NFC]) versus internet-based methods (eg, web-based cloud services) without anonymization. The second comparison evaluated comfort levels in sharing fully anonymized data with public health authorities versus partially anonymized data intended for public safety purposes. These nonparametric tests were chosen due to the ordinal nature of the survey data and the non-normal distribution of differences between paired responses. A chi-square test of independence was used to evaluate for differences in data sharing comfort between national health authorities, international health authorities, and private companies.

### Ethical Considerations

The study protocol [27] underwent peer review and was published in an academic journal. The study involves human participants and received ethical approval from the Social Research Ethics Committee, University College Cork, Ireland (SREC/SOM/21062023/2). Participants provided informed consent to participate in the study, participation was voluntary, and the participants could withdraw at any time. The data collected were anonymized, and no personal identifiers were collected. No compensation or incentives were provided for participation.

## Results

### Demographics

A total of 273 survey responses were collected, 198 responses were completed to the end of the continuous use section, and 169 responses were completed in their entirety. Participants identified as 52.7% (144/273) male, 45.8% (125/273) female, and 1.5% (4/273) other. The age of participants ranged from 18 to 65 years, with a mean age of 35.6 (SD 11.7) years.

### Validity and Reliability of the Model

Table 1 shows that the construct reliability of the measurement model was satisfactory, as composite reliability and Cronbach α coefficients are greater than 0.7 (except for confirmation). Bootstrapping the results showed *P*<.001 for all the indices in Table 1. Similarly, the model exhibited strong reliability, with all the loadings (in bold) greater than 0.7 except for USAB 1 (usability) and USAB 2. However, USAB 1 and USAB 2 were kept in the model because the loadings were still greater than 0.4.

The average variance extracted for all constructs was greater than 0.5, indicating good convergent validity (Table 1). The discriminant validity of the model was satisfactory, as the square roots of average variance extracted (in bold) are greater than the correlations (Table 1). The higher loadings of the indicators (in bold) compared with the cross-loadings also support the discriminant validity of the model (Table 2).

### Factors Influencing Continuous Use

**Figure 2.**
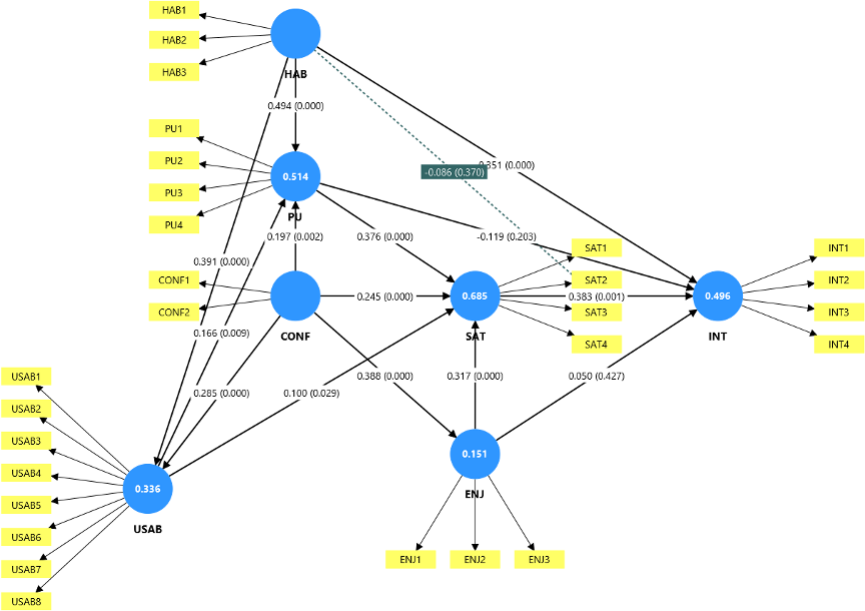
Path diagram for continuous smartwatch use based on the Expectation-Confirmation Model. The number within each circular shape indicates the *R*^2^ value for each construct. The numbers on the connecting arrows represent the path coefficients, with the corresponding *P* values in parentheses. CONF: confirmation; ENJ: enjoyment; HAB: habit; INT: continuance intention; PU: perceived usability; SAT: satisfaction; USAB: usability.

The path diagram (Figure 2) shows the *R*^2^ values, which, translated to percentages, show that the model explained 15%, 34%, 50%, 51%, and 62% of the variation in perceived usability, perceived enjoyment, continuance intention, perceived usefulness, and satisfaction, respectively.

The path diagram indicates that 50% of the variation in continuance intention to use a smartwatch was explained through habit (*β*=.35; *P*<.001) and satisfaction (*β*=.38; *P*<.001). Although other constructs did not show a significant direct influence on continuance intention to use smartwatch, 69% of the variation in satisfaction is explained through perceived usefulness (*β*=.38; *P*<.001), perceived enjoyment (*β*=.32; *P*<.001), confirmation (*β*=.24; *P*<.001), and perceived usability (*β*=.10; *P*=.03). Thus, the result indicates that continuous use of smartwatches is explained by habit and satisfaction, which are in turn explained by perceived usefulness, perceived enjoyment, confirmation, and perceived usability. Refer to the questions corresponding to each of these constructs in Multimedia Appendix 1.

### Data Sharing Results

The analysis of data-sharing preferences indicated significant differences in comfort levels depending on the method of data transmission and the level of data anonymization. These are subcategorized in detail in the following subsections.

#### Internet- Versus Noninternet-Based Methods of Data Sharing With a Physician

The Wilcoxon signed-rank test was used to compare respondents’ comfort levels when sharing health data collected from their device with a physician using noninternet methods (eg, in the doctor’s office via wired connection, Bluetooth, and NFC) versus internet-based methods (eg, web-based cloud services) without anonymization. This is shown in Figure 2. The results indicated 8 negative ranks (mean rank=26.25; sum of ranks=210.00), 57 positive ranks (mean rank=33.95; sum of ranks=1935.00), and 104 ties. There was a statistically significant difference in comfort levels between the 2 sharing methods (*z*=–5.793, *P*<.001).

#### Partial Versus Complete Anonymization of Data

**Figure 3.**
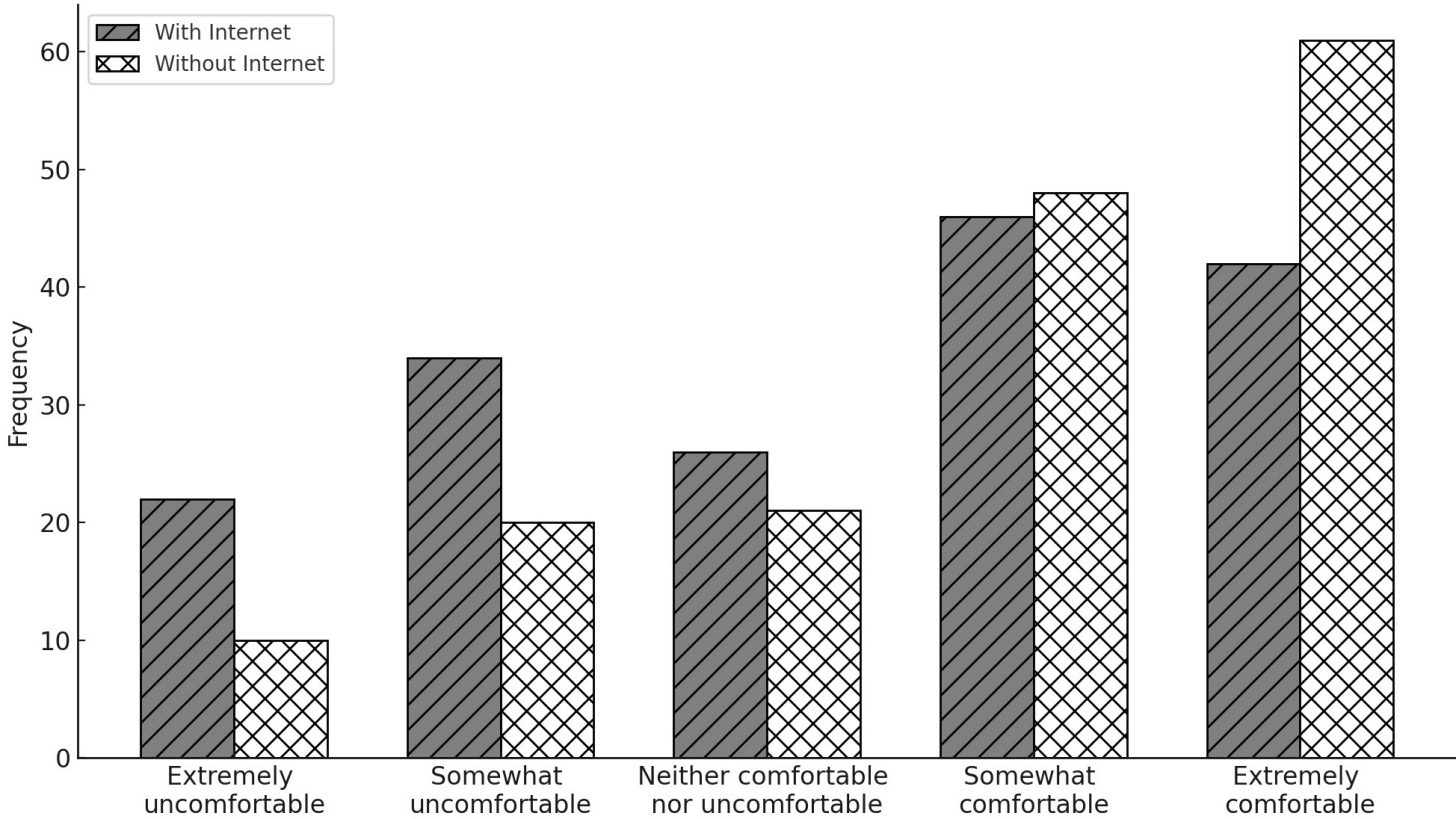
Comparison of comfort of sharing health data with a physician using internet versus noninternet methods.

The Wilcoxon signed-rank test was conducted to assess respondents’ comfort levels when sharing health data with public health authorities (eg, Health Service Executive [HSE], Ireland; National Health Service [NHS], United Kingdom; or the Center for Disease Control and Prevention [CDC], United States; and Health Canada) after full anonymization versus partial anonymization (eg, data used in public safety applications such as COVID-19 trackers that do not include names but may track precise locations). The test results showed 24 negative ranks (mean rank=34.92; sum of ranks=838.00), 54 positive ranks (mean rank=41.54; sum of ranks=2243.00), and 91 ties. The test statistic was *z*=−3.592 with a *P* value of less than .001, indicating a statistically significant difference in comfort levels between the 2 methods of data sharing as shown in Figure 3.

#### User Comfort Sharing Anonymized Data With Public Health Authorities and Private Companies

**Figure 4.**
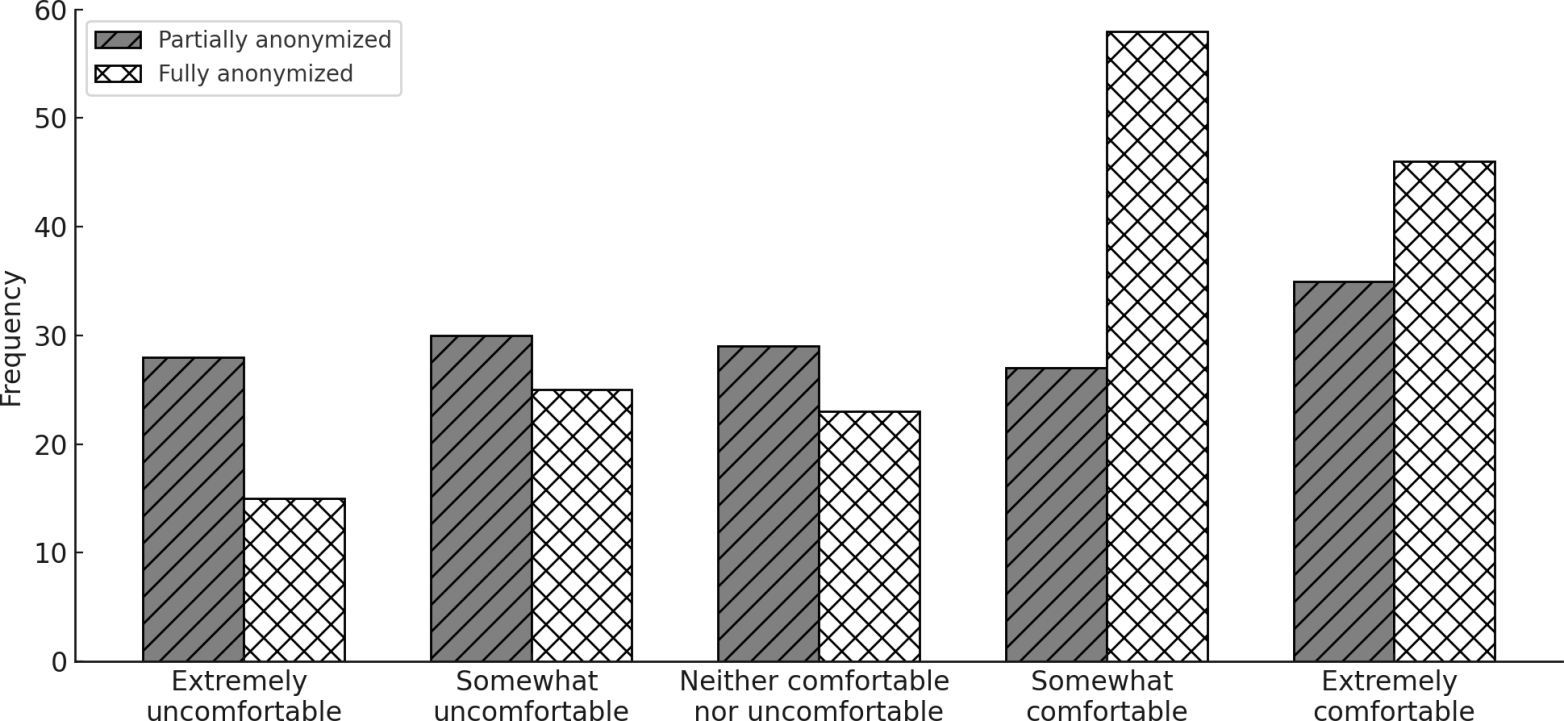
Comparison of comfort of sharing health data with a public health authority using fully versus partially anonymized data.

In Figure 4, the clear patterns of users’ preference in sharing anonymous data with different groups are that concerns are held with private companies much more so than either national or international health authorities. A chi-square test of independence was performed to evaluate the association between comfort with sharing data and the receiving bodies. There was no significant difference between the international and national health authorities, but there was between both of those groups and private companies (*P*<.001).

**Figure 5.**
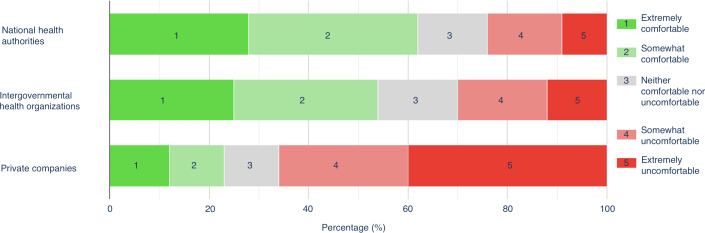
Comparison of comfort sharing data between intergovernmental, national, and private organizations.

When asked regarding comfort level sharing at the national level (eg, HSE, NHS, and CDC), 62.1% (105/169) of respondents were comfortable, while 24.3% (41/169) were not. At an international level (eg, WHO), 53.8% (91/169) of respondents were comfortable, while 30.2% (51/169) were uncomfortable sharing anonymized health data from their devices. For private companies, only 23.2% (39/168) of respondents were comfortable, while 66.1% (111/168) were not (Figure 5).

#### User Confidence in the Application of Shared Data

Overall, the majority of users expressed confidence in the potential benefits of sharing smartwatch data with health care providers. 

When users were asked (n=169), “I have confidence in the notion that sharing a larger collection of data with a general practitioner could improve the quality of the health care I receive,” 37.9% (64/169) of participants reported feeling somewhat confident, and 28.4% (48/169) were extremely confident. Conversely, 19.5% (33/169) were neutral, while 10.1% (17/169) were somewhat unconfident, and 4.1% (7/169) were extremely unconfident. These results highlight a predominantly positive perception of data sharing, though a minority of users still harbor reservations.

## Discussion

### Principal Findings

#### Determinants of Continuous Intention to Use Smartwatches

Habit formation emerged as the most influential factor driving the continuous use of smartwatches. Users who successfully incorporated smartwatches into their daily routines were more likely to intend to continuously use their smartwatch, highlighting the critical role of habit in sustained use. This finding underscores the importance of designing features that support routine development, such as daily reminders, goal-setting mechanisms, and integration with existing lifestyle habits [28-30]. By facilitating habit formation, manufacturers and app developers can likely enhance user retention and promote long-term engagement with smartwatches, ultimately improving the effectiveness of these devices in supporting health, wellness, and longitudinal data collection.

User satisfaction with the device was identified as the second most significant factor influencing continuous use, reflecting the importance of a positive user experience in sustaining engagement. Users who were satisfied with their smartwatch’s performance, design, and features were more likely to maintain a regular intention to use. Enhancing satisfaction can be achieved through user-friendly interfaces, reliable functionality, and responsive customer support [31,32]. These elements help create a seamless experience that meets user expectations, reinforcing the perception of value and driving continued use. Addressing user feedback and providing regular and useful updates to resolve issues can further enhance satisfaction [33], ensuring that the device remains relevant and effective over time.

Perceived enjoyment of using the device played a substantial role in influencing satisfaction. Users who found pleasure in using their smartwatches were more likely to derive satisfaction from using them, which likely encouraged more consistent engagement with the devices. To capitalize on this, developers should focus on creating engaging and user-engaging features, such as gamification, social sharing, and personalized feedback, which can enhance the overall user experience [34,35]. By making the interaction with the device enjoyable, users are more likely to view the smartwatch as a rewarding and integral part of their daily routine, thus promoting sustained use.

Perceived usefulness was found to have a moderate impact on continuous use, indicating that while users value the practical benefits of their smartwatches, it is not the primary driver of sustained engagement. Users who believed that their smartwatch provided valuable health insights and practical support in daily activities were more inclined to maintain regular use [36]. To enhance perceived usefulness, developers should emphasize features that deliver clear, actionable health information and demonstrate the tangible benefits of smartwatch use [37], such as tracking health goals, monitoring chronic conditions, and providing timely health alerts.

#### Smartwatch Health Data Sharing

The study revealed that users are significantly more comfortable sharing health data with physicians through noninternet methods, such as Bluetooth or NFC, compared with internet-based methods such as cloud services. This preference underscores the concerns users have regarding online data security and privacy. The findings suggest a need for health care providers and technology developers to offer secure offline data-sharing options to meet user comfort levels and build trust. Enhancing user education around the security of online data-sharing methods could also help address these privacy concerns and improve user confidence.

The preference for fully anonymized data over partially anonymized data when sharing with public health authorities was also evident. Users expressed higher comfort levels with data that are completely anonymized, indicating a strong desire for privacy and control over personal information. This finding emphasizes the importance of implementing robust anonymization protocols in public health data-sharing practices. However, achieving anonymized data in practice remains a daunting task, and medical researchers themselves question when data are truly anonymized in line with data protection regulations [38]. Such concerns are justified given that a recent statistical model found that 99.98% of Americans could be reidentified using 15 demographic attributes from a deidentified dataset [39]. Nonetheless, there have been numerous advances in data-sharing techniques aimed at preserving patient privacy. For instance, personal health trains based on federated learning do not require the centralization of data for analysis [40], preventing the creation of large, all-encompassing databases over individuals. Although not always used with anonymized data, such advancements in data privacy may be able to garner more trust in data sharing with public health authorities. Transparent communication about how anonymized data are used and the measures taken to protect user privacy could further encourage participation in public health initiatives that rely on data sharing [41,42].

The analysis revealed a generally positive outlook on the potential benefits of sharing smartwatch data with health care providers. Overall, 66.3% (112/169) of users reported confidence that sharing their data with their treating physician could improve health care quality. This reflects a strong belief among users in the use of these devices in medical contexts. However, a notable minority of users remained neutral (19.5%, 33/169) or expressed a lack of confidence (14.2%, 24/169), suggesting that there are still reservations about the effectiveness and security of data integration into clinical practice.

These mixed confidence levels suggest that while many users are optimistic, there is still uncertainty about the practical impact of sharing smartwatch data on health care outcomes. The variability in responses underscores the importance of further studies to rigorously evaluate the benefits and address user concerns. Establishing evidence-based practices, alongside clear communication from health care providers about data handling, security, and potential advantages, may help to build greater trust and encourage more widespread data-sharing between users and interested treating physicians [43,44].

### Comparison With Previous Work

Given the growing body of literature examining smartwatches and their use in health care, we aimed to expand this base knowledge to better understand the determinants of continuous intention and data sharing from the smartwatch user’s perspective. The findings of this study align with previous literature emphasizing the importance of habit formation, satisfaction, enjoyment, and perceived usefulness in driving technology adoption and sustained engagement [6-8]. Previous studies have consistently highlighted that habit formation plays a crucial role in the long-term use of health technologies, reinforcing our results that daily integration and routine use significantly boost engagement. Novel findings in our study include understanding that the use of internet-based data sharing methods can lead to users feeling less comfortable sharing data. This, in addition to the other findings related to data sharing with different levels of organizations, helps fill in gaps in the literature.

### Limitations and Future Directions

This study has limitations. First, the reliance on self-reported data introduces potential biases, including social desirability bias, where participants may overstate their engagement or comfort with data-sharing practices. Second, the convenience sampling approach may not fully capture the broader diversity of smartwatch users, skewing results toward more tech-savvy or health-conscious individuals through self-selection bias. Future studies could use alternative sampling models, such as stratified sampling, to account for this.

The cross-sectional design captures attitudes at a single point in time, limiting insights into how preferences and behaviors might change over time, particularly as technology and privacy practices evolve. Future work could include longitudinal studies to address this.

Further, to the knowledge of the authors, no existing, validated questionnaire collects the specific outcomes deemed pertinent to the data sharing subsection of this study. Development of new questionnaires is a time-intensive and rigorous process and typically requires multiple evaluations to ensure validity and reliability. Drawing on the broad expertise of the research team, the current questionnaire was developed in line with the primary aims of this study. This questionnaire did undergo peer review [27], giving credence to its face validity. However, further psychometric evaluations would be recommended to test the inherent properties of this instrument before its broad uptake.

This study did not differentiate between smartwatch brands, operating systems, or regional data legislation. This may be addressed in future research with a larger sample size or more restrictive inclusion criteria. In addition, as features continue to emerge, particularly artificial intelligence–based features, evaluating their impact on continuous intention and data-sharing preferences is warranted.

The study did not differentiate between various data-sharing scenarios, such as differences in security features or types of health data being shared, which could influence user comfort levels. Future research should further explore these nuances to better understand what drives trust in data sharing through qualitative methodologies such as focus groups or interviews. Of note, there may be geographic variations in preference due to factors such as private health care; these could be investigated in future research. Finally, while the study provides valuable insights into user perceptions, further research is needed to establish the actual clinical impact of smartwatch data integration on health care outcomes.

The relationship between the individual constructs of the ECM and continuous intention and comfort with data sharing was not addressed in our survey. This could be evaluated in future work.

### Conclusions

To successfully integrate smartwatch data into clinical care, designing features that support habit-building, enhance user satisfaction, and create enjoyable experiences are necessary for sustained engagement. Furthermore, the preference for noninternet data-sharing methods and fully anonymized data reflects ongoing user concerns about privacy and security, indicating a need for robust and transparent data-sharing practices. Users were more inclined to share data with physicians and health organizations than with private companies, while generally expressing confidence in the potential of smartwatch data to improve health care. By aligning device features and data-sharing protocols with user preferences, manufacturers, health care providers, and policy makers can enhance user engagement and maximize the potential of smartwatches to support individual health management and public health initiatives.

## Supplementary material

10.2196/67414 Multimedia Appendix 1 Online survey developed on the platform Qualtrics.

10.2196/67414 Checklist 1 STROBE checklist.

## Abbreviations

CDC: Center for Disease Control and Prevention
ECM: Expectation-Confirmation Model
HSE: Health Service Executive
NFC: near-field communication
NHS: National Health Service
SEM: structural equation modeling
STROBE: Strengthening the Reporting of Observational Studies in Epidemiology
