# Supplementary material for: Determinants of Continuous Smartwatch Use and Data-Sharing Preferences With Physicians, Public Health Authorities, and Private Companies: Cross-Sectional Survey of Smartwatch Users
Source: J Med Internet Res. 2025 Aug 18;27:e67414. doi: 10.2196/67414 (PMC12360733; doi:10.2196/67414)
Supplement: Multimedia Appendix 1 [file jmir-v27-e67414-s001.docx]

CLEAR Wearable Technologies Ireland WEAR&SHARE

Survey Flow

Standard: Eligibility (2 Questions)

Standard: Participant Information (1 Question)

Standard: Data Protection Policy (1 Question)

Standard: Demographics (2 Questions)

Block: Wear (28 Questions)

Standard: Share (10 Questions)

Standard: Focus Group Interest (1 Question)

| Page Break |  |
| --- | --- |

Start of Block: Eligibility

E1 I am over 18 years of age.

- Yes (please continue) (1)
- No (please exit survey) (2)

E2 I have currently or have previously used a smart watch.

- Yes (please continue) (1)
- No (please exit survey) (2)

End of Block: Eligibility

Start of Block: Participant Information

Information
   

 **Information for Participants**


 Your participation in this study is voluntary and you can withdraw at any time.

 If you agree to participate, you will be prompted to confirm that you understand and are aware of the ways in which the data you provide will be used and stored.

 *Here are the answers to some common questions that you may have:*

 **Will my information be kept confidential?**
 Yes, all information gathered during this study will be anonymous and cannot be linked to you. Only the researchers and statisticians will be able to access the data.

 **What will happen to the information I give?**
 The data will be kept confidential for the duration of the study. It will be securely stored as a password-protected, encrypted file. Upon completion of the study, the data will be retained for a minimum of ten years and then will be destroyed.
*N.B. You can request to have your information withdrawn from the study by emailing Anthony Goodings (120105136@umail.ucc.ie) within two weeks of completing the study.*

 **What will happen to the results?**
 The study will be submitted for publication in a peer-reviewed medical journal.

 **What are the possible disadvantages of taking part?**
 This project aims to answer a small number of research questions regarding an emotionally neutral topic, there are no foreseen disadvantages.

 **What if I encounter a problem?**
 At the end of the survey, there will be a number of questions that will allow you to express how you are feeling and your experience with the survey. If you have any further concerns, comments, or questions you may contact:

 Lead Researcher
 Anthony J. Goodings
 120105136@umail.ucc.ie
Fourth-Year Medical Student
 University College Cork
 Ireland

Principal Investigator
Dr. John O'Donoghue
UCC ASSERT Centre

|  | I understand and consent (1) | I do not consent (2) |
| --- | --- | --- |
| I understand that my data will be collected and stored anonymously. (4) |  |  |
| I understand that I can withdraw at anytime by leaving this page. (5) |  |  |
| I understand that I can ask for my data to be withdrawn within two weeks of completing the study by emailing 120105136@umail.ucc.ie (6) |  |  |

End of Block: Participant Information

Start of Block: Data Protection Policy

Handling of Data If you have any queries about this research project, you can contact Anthony Goodings at 120105136@umail.ucc.ie or contact the principal investigator involved in this project through the email john.odonoghue@ucc.ie.

 If you have a concern about how we have handled your personal data, you are entitled to this raise this with the Data Protection Commission: https://www.dataprotection.ie/.

 “A personal data breach occurs when the data is accessed, disclosed, altered, lost or destroyed in contravention of an organisation’s obligation to keep personal data in its possession safe and secure”
 https://www.dataprotection.ie/ UCC'S Data Protection Officer (DPO) is Catriona O'Sullivan, Information Compliance Manager, University College Cork, 4 Carrigside, College Road, Cork, Ireland. Telephone: +353 (0)21 4903949* Email: gdpr@ucc.ie

 The Data Controller for this study is Dr. John O'Donoghue (john.odonoghue@ucc.ie). If you have a complaint about how this research was conducted please contact via email, the Social Science Research Ethics Committee (SREC) at: srec@ucc.ie.

 **Data Protection Notice**
 At University College Cork, we treat your privacy seriously. Any personal data which you provide to the University will be treated with the highest standards of security and confidentiality, in accordance with Irish and European Data Protection legislation. This notice sets out details of the information that we collect, how we process it and who we share it with. It also explains your rights under data protection law in relation to our processing of your data.

 Who we are
 Throughout this Notice, “we”, “us” and “our” refers to University College Cork, as study sponsor. For more information about us, please refer to our website: www.ucc.ie

 How we will use your personal data
 By participating in the study, information from you (also called “personal data”) will be collected for the study purposes mentioned in the Participant Information Leaflet above. This personal data may include, for example: information that directly identifies you (such as your name, and your year of birth) your gender, ethnic and racial background information on your health and medical condition including your medical history your treatments and your response to treatments information contained in your blood samples and the results after analysis Personal data collected at any time during the study will be kept strictly confidential. To ensure confidentiality, the data generated during the study is coded with a number that will identify you in the study. Any information that leaves the clinical site will be labelled with your code instead of your name.

 Data that directly identifies you (uncoded data) is stored securely by the Chief Investigator. A list or ‘key’ linking your study number to your name will be kept stored securely (locked cupboard in a room with restricted access) by the Chief Investigator for 10 years & destroyed thereafter. The study team who access to your uncoded data are subject to professional secrecy and confidentiality.

 Who will access my personal data?
 Your uncoded data will only be accessible to the study investigator and study staff. Results of the study will be provided to the Clinical Research Ethics Committee of the Cork Teaching Hospitals (CREC) in compliance with national and international regulations on clinical studies.

 The purpose and legal basis for collecting your data Any personal data you provide to us during the course of this study will be processed fairly and lawfully. Signing the Informed Consent Form means that your personal data will be used for the purposes outlined in the Participant Information Leaflet. Personal data collected during this study and the results of the study may be presented for scientific purposes. However, you will never be identified individually during these presentations. Your identity will not be revealed in any reports or publications. The clinical site and the investigator will use your personal data within the scope defined above. The General Data Protection Regulation allows us to process your data because you have provided your consent. You are entitled to withdraw your consent at any time.

 How long we will keep your data
 The personal data collected in the study will be kept for a period of 10 years after the end of the study. Thereafter, they may be stored for a further period of time for legal reasons (e.g. revised retention obligations), or more if required by law.

 Your rights
 You have various rights under data protection law, subject to certain exemptions, in connection with our processing of your personal data, including the right: to find out if we use your personal data, access your personal data and receive copies of your personal data. to have inaccurate/incomplete information corrected and updated. in certain circumstances, to have your details deleted from systems that we use to process your personal data or have the use of your personal data restricted in certain ways. to object to certain processing of your data by UCC. to exercise your right to data portability where applicable (i.e. obtain a copy of your personal data in a commonly used electronic form. to withdraw your consent to the processing of your data at any time without giving a reason by notifying your decision to the investigator. This will not affect the lawfulness of processing data about you based on your consent before the withdrawal. If you withdraw your consent for data processing, your participation in the study stops and no further data will be collected from you. Your study physician will present you the options you have concerning your personal data. Along with study withdrawal, you have the right to request the deletion of data about you if your data are no longer necessary for the purposes of processing or there is no other legal ground for their further processing.

|  | Yes (1) | No (2) |
| --- | --- | --- |
| I have read the data protection notice and consent to proceed. (1) |  |  |

End of Block: Data Protection Policy

Start of Block: Demographics

A Please write your age.

________________________________________________________________

G Please select the gender you identify with.

- Female (1)
- Male (2)
- Other (3)

End of Block: Demographics

Start of Block: Wear

CONF1 My experience with using the smartwatch is better than what I expected.

- (1) Much worse than expected (1)
- (2) Somewhat worse than expected (2)
- (3) Slightly worse than expected (3)
- (4) As good as expected (4)
- (5) Slightly better than expected (5)
- (6) Somewhat better than expected (6)
- (7) Much better than expected (7)

CONF2 The expectations that I had of the smartwatch before using it were confirmed.

- (1) Entirely disagree (1)
- (2) Somewhat disagree (2)
- (3) Slightly disagree (3)
- (4) Neutral (4)
- (5) Slightly agree (5)
- (6) Somewhat agree (6)
- (7) Entirely agree (7)

PU1 I find the smartwatch useful in my daily life.

- (1) Entirely disagree (1)
- (2) Somewhat disagree (2)
- (3) Slightly disagree (3)
- (4) Neutral (4)
- (5) Slightly agree (5)
- (6) Somewhat agree (6)
- (7) Entirely agree (7)

PU2 Using the smartwatch helps me accomplish things more quickly.

- (1) Entirely disagree (1)
- (2) Somewhat disagree (2)
- (3) Slightly disagree (3)
- (4) Neutral (4)
- (5) Slightly agree (5)
- (6) Somewhat agree (6)
- (7) Entirely agree (7)

PU3 Using the smartwatch increases my productivity.

- (1) Entirely disagree (1)
- (2) Somewhat disagree (2)
- (3) Slightly disagree (3)
- (4) Neutral (4)
- (5) Slightly agree (5)
- (6) Somewhat agree (6)
- (7) Entirely agree (7)

PU4 Using the smartwatch helps me to perform many things more conveniently.

- (1) Entirely disagree (1)
- (2) Somewhat disagree (2)
- (3) Slightly disagree (3)
- (4) Neutral (4)
- (5) Slightly agree (5)
- (6) Somewhat agree (6)
- (7) Entirely agree (7)

SAT1 How satisfied are you with your overall experience of smartwatch use?

- (1) Very dissatisfied (1)
- (2) Somewhat disatisfied (2)
- (3) Slightly disatisfied (3)
- (4) Neutral (4)
- (5) Slightly satisfied (5)
- (6) Somewhat satisfied (6)
- (7) Very satisfied (7)

SAT2 How pleasing did you find your overall experience of smartwatch use?

- (1) Very displeasing (1)
- (2) Somewhat displeasing (2)
- (3) Slightly disapleasing (3)
- (4) Neutral (4)
- (5) Slightly pleasing (5)
- (6) Somewhat pleasing (6)
- (7) Very pleasing (7)

SAT3 How did your experience regarding the ease of use of the smartwatch leave you feeling?

- (1) Very frustrated (1)
- (2) Somewhat frustrated (2)
- (3) Slightly frustrated (3)
- (4) Neutral (4)
- (5) Slightly contented (5)
- (6) Somewhat contented (6)
- (7) Very contented (7)

SAT4 How was your overall experience of smartwatch use?

- (1) Absolutely terrible (1)
- (2) Very poor (2)
- (3) Somewhat poor (3)
- (4) Neutral (4)
- (5) Somewhat good (5)
- (6) Very good (6)
- (7) Absolutely delighted (7)

HAB1 Using the smartwatch has become automatic to me.

- (1) Entirely disagree (1)
- (2) Somewhat disagree (2)
- (3) Slightly disagree (3)
- (4) Neutral (4)
- (5) Slightly agree (5)
- (6) Somewhat agree (6)
- (7) Entirely agree (7)

HAB2 Using the smartwatch is natural to me.

- (1) Entirely disagree (1)
- (2) Somewhat disagree (2)
- (3) Slightly disagree (3)
- (4) Neutral (4)
- (5) Slightly agree (5)
- (6) Somewhat agree (6)
- (7) Entirely agree (7)

HAB3 When faced with a particular task, using the smartwatch is an obvious choice for me.

- (1) Entirely disagree (1)
- (2) Somewhat disagree (2)
- (3) Slightly disagree (3)
- (4) Neutral (4)
- (5) Slightly agree (5)
- (6) Somewhat agree (6)
- (7) Entirely agree (7)

USAB1 Every feature and function in the smartwatch is easy to understand.

- (1) Entirely disagree (1)
- (2) Somewhat disagree (2)
- (3) Slightly disagree (3)
- (4) Neutral (4)
- (5) Slightly agree (5)
- (6) Somewhat agree (6)
- (7) Entirely agree (7)

USAB2 The smartwatch is simple to use, even when using it for the first time.

- (1) Entirely disagree (1)
- (2) Somewhat disagree (2)
- (3) Slightly disagree (3)
- (4) Neutral (4)
- (5) Slightly agree (5)
- (6) Somewhat agree (6)
- (7) Entirely agree (7)

USAB3 The contents of the smartwatch are organized in such a way that makes it easy for me to know where I am when navigating it.

- (1) Entirely disagree (1)
- (2) Somewhat disagree (2)
- (3) Slightly disagree (3)
- (4) Neutral (4)
- (5) Slightly agree (5)
- (6) Somewhat agree (6)
- (7) Entirely agree (7)

USAB4 The amount of information displayed in the smartwatch is appropriate.

- (1) Entirely disagree (1)
- (2) Somewhat disagree (2)
- (3) Slightly disagree (3)
- (4) Neutral (4)
- (5) Slightly agree (5)
- (6) Somewhat agree (6)
- (7) Entirely agree (7)

USAB5 Searching and checking the information that I need from the smartwatch is quick.

- (1) Entirely disagree (1)
- (2) Somewhat disagree (2)
- (3) Slightly disagree (3)
- (4) Neutral (4)
- (5) Slightly agree (5)
- (6) Somewhat agree (6)
- (7) Entirely agree (7)

USAB6 It is easy to find the information I need from the smartwatch.

- (1) Entirely disagree (1)
- (2) Somewhat disagree (2)
- (3) Slightly disagree (3)
- (4) Neutral (4)
- (5) Slightly agree (5)
- (6) Somewhat agree (6)
- (7) Entirely agree (7)

USAB7 It is easy to find the functions I need from the smartwatch.

- (1) Entirely disagree (1)
- (2) Somewhat disagree (2)
- (3) Slightly disagree (3)
- (4) Neutral (4)
- (5) Slightly agree (5)
- (6) Somewhat agree (6)
- (7) Entirely agree (7)

USAB8 The smartwatch provides accurate information and functions that I need.

- (1) Entirely disagree (1)
- (2) Somewhat disagree (2)
- (3) Slightly disagree (3)
- (4) Neutral (4)
- (5) Slightly agree (5)
- (6) Somewhat agree (6)
- (7) Entirely agree (7)

| Page Break |  |
| --- | --- |

ENJ1 I have fun interacting with the smartwatch.

- (1) Entirely disagree (1)
- (2) Somewhat disagree (2)
- (3) Slightly disagree (3)
- (4) Neutral (4)
- (5) Slightly agree (5)
- (6) Somewhat agree (6)
- (7) Entirely agree (7)

ENJ2 Using the smartwatch provides me with a lot of enjoyment.

- (1) Entirely disagree (1)
- (2) Somewhat disagree (2)
- (3) Slightly disagree (3)
- (4) Neutral (4)
- (5) Slightly agree (5)
- (6) Somewhat agree (6)
- (7) Entirely agree (7)

ENJ3 I enjoy using the smartwatch.

- (1) Entirely disagree (1)
- (2) Somewhat disagree (2)
- (3) Slightly disagree (3)
- (4) Neutral (4)
- (5) Slightly agree (5)
- (6) Somewhat agree (6)
- (7) Entirely agree (7)

INT1 I intend to continue using the smartwatch, rather than discontinue its use.

- (1) Entirely disagree (1)
- (2) Somewhat disagree (2)
- (3) Slightly disagree (3)
- (4) Neutral (4)
- (5) Slightly agree (5)
- (6) Somewhat agree (6)
- (7) Entirely agree (7)

INT2 I plan to continue using the smartwatch.

- (1) Entirely disagree (1)
- (2) Somewhat disagree (2)
- (3) Slightly disagree (3)
- (4) Neutral (4)
- (5) Slightly agree (5)
- (6) Somewhat agree (6)
- (7) Entirely agree (7)

INT3 I will continue using the smartwatch.

- (1) Entirely disagree (1)
- (2) Somewhat disagree (2)
- (3) Slightly disagree (3)
- (4) Neutral (4)
- (5) Slightly agree (5)
- (6) Somewhat agree (6)
- (7) Entirely agree (7)

INT4 I predict I will continue using the smartwatch in the future.

- (1) Entirely disagree (1)
- (2) Somewhat disagree (2)
- (3) Slightly disagree (3)
- (4) Neutral (4)
- (5) Slightly agree (5)
- (6) Somewhat agree (6)
- (7) Entirely agree (7)

End of Block: Wear

Start of Block: Share

S1 I am comfortable with the health data collected from my device being stored on a cloud.

- Extremely uncomfortable (1)
- Somewhat uncomfortable (2)
- Neither comfortable nor uncomfortable (3)
- Somewhat comfortable (4)
- Extremely comfortable (5)

S2 I am comfortable with the health data collected from my device being shared with a physician using a method involving the internet (e.g. cloud) without being anonymized.

- Extremely uncomfortable (1)
- Somewhat uncomfortable (2)
- Neither comfortable nor uncomfortable (3)
- Somewhat comfortable (4)
- Extremely comfortable (5)

S3 I am comfortable with the health data collected from my device being shared with a physician using a method not involving the internet (e.g. in the doctor’s office via wired connection, bluetooth, NFC) without being anonymized.

- Extremely uncomfortable (1)
- Somewhat uncomfortable (2)
- Neither comfortable nor uncomfortable (3)
- Somewhat comfortable (4)
- Extremely comfortable (5)

S4 I am comfortable with the health data collected from my device being shared with a governmental health authority (e.g. HSE, NHS, CDC, Health Canada) after being anonymized.

- Extremely uncomfortable (1)
- Somewhat uncomfortable (2)
- Neither comfortable nor uncomfortable (3)
- Somewhat comfortable (4)
- Extremely comfortable (5)

S5 I am comfortable with the health data from my device being shared with other governmental departments (e.g. CSO, ONS, USCB, Statistics Canada) after being anonymized.

- Extremely uncomfortable (1)
- Somewhat uncomfortable (2)
- Neither comfortable nor uncomfortable (3)
- Somewhat comfortable (4)
- Extremely comfortable (5)

S6 I am comfortable with the health data from my device being shared with intergovernmental health organizations (e.g. WHO) after being anonymized.

- Extremely uncomfortable (1)
- Somewhat uncomfortable (2)
- Neither comfortable nor uncomfortable (3)
- Somewhat comfortable (4)
- Extremely comfortable (5)

S7 I am comfortable with my health data being shared with private companies after being anonymized.

- Extremely uncomfortable (1)
- Somewhat uncomfortable (2)
- Neither comfortable nor uncomfortable (3)
- Somewhat comfortable (4)
- Extremely comfortable (5)

S8 I am comfortable with my health data being shared with governmental health authorities (e.g. HSE, NHS, CDC, Health Canada) after being partially anonymized with the goal of ensuring better public safety (e.g. COVID-19 tracker apps that track your precise location but do not share your name or other personal details).

- Extremely uncomfortable (1)
- Somewhat uncomfortable (2)
- Neither comfortable nor uncomfortable (3)
- Somewhat comfortable (4)
- Extremely comfortable (5)

S9 I have confidence in private tech companies to keep my data private (e.g. not selling data to advertisers).

- Extremely unconfident (1)
- Somewhat unconfident (2)
- Neither confident nor unconfident (3)
- Somewhat confident (4)
- Extremely confident (5)

S10 I have confidence in the notion that sharing a larger collection of data with a general practitioner could improve the quality of the health care I receive.

- Extremely unconfident (1)
- Somewhat unconfident (2)
- Neither confident nor unconfident (3)
- Somewhat confident (4)
- Extremely confident (5)

End of Block: Share

Start of Block: Focus Group Interest

Contact If you would like to be contacted regarding possible participation in a focus group, please leave your email below. If not, please leave blank or type n/a.

________________________________________________________________

End of Block: Focus Group Interest
